# Prevalence, incidence, and treatment of anaemia in patients with non-dialysis-dependent chronic kidney disease: findings from a retrospective real-world study in Italy

**DOI:** 10.1007/s40620-022-01475-x

**Published:** 2022-11-12

**Authors:** Roberto Minutolo, Giuseppe Grandaliano, Paolo Di Rienzo, Robert Snijder, Luca Degli Esposti, Valentina Perrone, Lora Todorova

**Affiliations:** 1 Nephrology Division, Department of Advanced Medical and Surgical Sciences, University of Campania Luigi Vanvitelli, Naples, Italy; 2 Nephrology Unit, Department of Translational Medicine and Surgery, Università Cattolica del Sacro Cuore, Rome, Italy; 3 Department of Medical and Surgical Sciences, Fondazione Policlinico Universitario A. Gemelli IRCCS, Rome, Italy; 4 Astellas Pharma Italia S.P.A., Milan, Italy; 5 Astellas Pharma Europe Ltd, Leiden, Netherlands; 6 Economics & Outcomes Research, CliCon Srl Health, Bologna, Italy; 7 Astellas Pharma Inc., Addlestone, UK

**Keywords:** Anaemia, Epidemiology, ESA, Non-dialysis-dependent CKD, Real world, Retrospective

## Abstract

**Background:**

Limited data are available on the epidemiology and clinical management of anaemia in patients with non-dialysis-dependent chronic kidney disease (NDD-CKD).

**Methods:**

This retrospective observational study was based on records from databases of five Local Health Units across Italy. Adults with reported NDD-CKD stage 3a–5 between 1 January 2014 and 31 December 2016 were identified. Annual prevalence and incidence of anaemia (age- and sex-standardised) and clinical management (erythropoiesis-stimulating agents [ESAs], intravenous [IV] iron, and blood transfusions) were evaluated. Eligibility for ESAs was defined by ≥ 2 records of Hb < 10 g/dL, or < 11 g/dL over 6 months.

**Results:**

Overall, 101,143 individuals with NDD-CKD (3a–5) recorded between 2014 and 2016 were identified, of whom 40,020 (39.6%) were anaemic. Prevalence of anaemia was 33.8% in 2016 and incidence of anaemia was stable (11.4–12.4%) from 2014 to 2016. Prevalence and incidence of anaemia increased with CKD stage. Among eligible patients, 12.8% with Hb < 11 g/dL and 15.5% with Hb < 10 g/dL received ESAs, and the proportion treated increased with CKD stage. Among ESA-treated patients with at least 2 years of follow up, 18.4% and 19.3% received IV iron in the Hb < 11 and < 10 g/dL groups, respectively, and 16.5% and 19.4% received blood transfusions. Corresponding proportions for the overall anaemic cohort were 9.0% and 11.3%, respectively.

**Conclusions:**

Anaemia is a significant issue in patients with NDD-CKD. Low rates of ESA treatment indicate a potential treatment gap and suggest that anaemia may not be adequately controlled in many patients.

**Graphical abstract:**

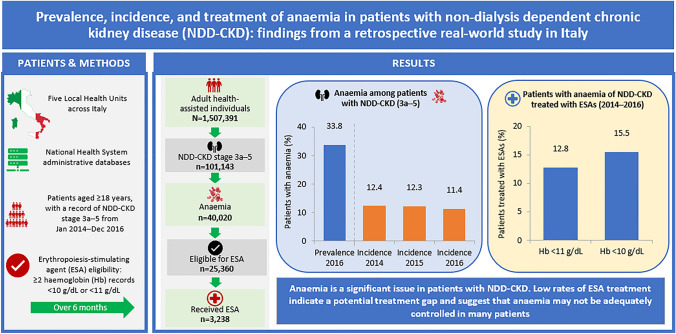

**Supplementary Information:**

The online version contains supplementary material available at 10.1007/s40620-022-01475-x.

## Introduction

Chronic kidney disease (CKD) has an estimated global prevalence of 11–13% and is a growing public health issue worldwide [1]. Data from the 2008–2012 Cardiovascular risk profile in Renal patients of the Italian Health Examination Survey (CARHES), which examined Italian residents aged 35–79 years, showed a CKD prevalence of about 7%, with early stages (1 and 2) accounting for nearly 60% of cases [2].

Anaemia is a common complication in patients with CKD, and its prevalence increases with advancing CKD stage [3, 4]. The main cause of anaemia associated with CKD is reduced production of erythropoietin by the failing kidneys as a result of impaired oxygen-sensing mechanisms in CKD [5]. Anaemia is associated with CKD progression and other adverse outcomes, including major adverse cardiovascular events, hospitalisation, and all-cause mortality [3].

Iron supplementation and erythropoiesis-stimulating agents (ESAs) are the mainstays of treatment for anaemia in CKD [6–8]. Recent data from the prospective, multinational Chronic Kidney Disease Outcomes and Practice Patterns Study (CKDopps) on the management of anaemia in patients with non-dialysis-dependent CKD (NDD-CKD) under nephrologist care in Brazil, France, Germany, and the USA, highlighted that anaemia monitoring and treatment are suboptimal, with many patients untreated despite guideline-based indications to treat [9, 10]. With respect to Italy, data on the prevalence and management of anaemia of NDD-CKD are limited. In a prospective cohort study of adults with NDD-CKD stage 3–5 attending 25 renal clinics in Italy in 2003, the prevalence of mild anaemia was 41.3% at the first visit [11]. Another prospective study in 19 outpatient renal clinics across Italy reported an unexpectedly high prevalence of anaemia (severe 18.0–19.3%, mild 43.2–44.0%) among patients with NDD-CKD over a 6-month period, which was linked to clinical inertia in initiating anaemia treatment [12].

Therefore, this observational study was designed to evaluate the prevalence of NDD-CKD (stage 3a–5), in addition to the prevalence and incidence of anaemia of NDD-CKD (stage 3a–5), and the therapeutic management of this complication in Italian clinical practice.

## Materials and methods

This was a retrospective cohort study involving adult patients (aged ≥ 18 years) with reported NDD-CKD stage 3a–5 between 1 January 2014 and 31 December 2016 (the inclusion period). Records were extracted from administrative (demographic, hospitalisation, pharmaceutical, and outpatient specialist services) and laboratory databases of five Local Health Units (LHUs) across Italy (full details provided in Online Resource 1). The Ethics Committee of each participating LHU was notified of this study and provided approval (reference numbers: Prot. N AslVC.Farm.19.02, Prot. 96/SegCE/2019, Prot. N. 0,219,118, Prot. N. 03, and Prot. CEUR-2020-Os-029 [Online Resource 1]).

**Fig. 1:**
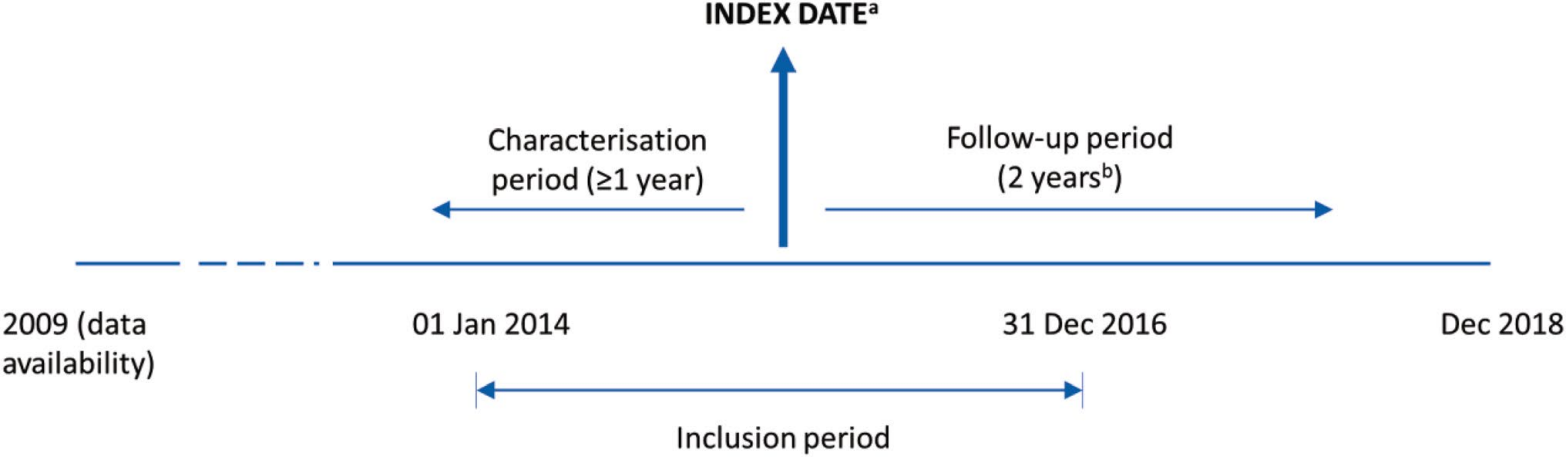
Study design. ^a^For the NDD−CKD cohort (CKD index date), date of first diagnosis of CKD stage 3a–5 or earliest record of eGFR <60 mL/min/1.73 m^2^ within the inclusion period; for the anaemic cohort (anaemia of CKD index date): date of earliest second of two consecutive tests (within 3 months of each other) reporting Hb <13 g/dL (males) or <12 g/dL (females) within the inclusion period; for the ESA−treated cohort (ESA treatment index date), date of first ESA prescription within the inclusion period. ^b^Or until death, dialysis, transplant, or exiting the database (whichever occurred first). *CKD* chronic kidney disease; *eGFR* estimated glomerular filtration rate; *ESA* erythropoiesis−stimulating agent; *Hb* haemoglobin; *NDD−CKD* non−dialysis dependent chronic kidney disease

NDD-CKD (3a–5) and respective stages were defined as detailed in Online Resource 2. Anaemia was defined as a haemoglobin (Hb) value below the cut-off specified by Kidney Disease Improving Global Outcomes (KDIGO) guidelines (< 13 g/dL in males and < 12 g/dL in females) [7]. Patients were grouped into three cohorts: NDD-CKD (3a–5) cohort, anaemia of NDD-CKD (3a–5) cohort, and anaemia of NDD-CKD (3a–5) ESA-treated cohort (referred to hereafter as NDD-CKD, anaemic, and ESA-treated cohorts, respectively). The three patient cohorts had distinct index dates during the study period (Fig. 1). Patients were excluded if 1) they were on dialysis at or before the CKD index date; 2) they had a cancer diagnosis ≤ 5 years before the CKD index date; 3) the characterisation (‘look-back’) period was < 1 year; 4) the anaemia index date was ≥ 1 month before the CKD index date (incident anaemic patients); or 5) the ESA treatment index date was ≥ 1 month prior to the anaemia index date (incident ESA-treated patients). For each cohort, the index date marked the beginning of the follow-up period (Fig. 1). An identification period of ≥ 1 year before the respective index dates was used to characterise patients in terms of comorbidities (see Online Resource 2 for details) and previous treatment for the management of anaemia. The follow-up period was 2 years after the respective index date, or from index date until death, dialysis, transplant, or exiting the database, whichever occurred first.

The objectives of this study were to: 1) estimate the annual prevalence of NDD-CKD; 2) estimate the annual prevalence and incidence of anaemia among patients with NDD-CKD; and 3) evaluate the management of anaemia in patients with NDD-CKD, including treatment with ESAs (frequency and types of ESAs used), intravenous iron, and blood transfusions.

Incident and prevalent patients within the three cohorts are defined in Online Resource 3. Results were age- and sex-standardised according to Italian census data from 1 January of the year following the inclusion date. Prevalence was defined as the presence of NDD-CKD or anaemia of NDD-CKD at 31 December of the year. Incident cases were defined as newly diagnosed NDD-CKD or anaemia of NDD-CKD during the year of interest. Eligibility for ESA treatment was defined using two Hb thresholds: ≥ 2 records of Hb < 10 g/dL over 6 months; and ≥ 2 records of Hb < 11 g/dL over 6 months. The Hb < 10 g/dL threshold is recommended by international guidelines [7], while the Hb < 11 g/dL threshold is supported by the Italian Society of Nephrology [13] and defined in the Italian Therapeutic Plan for ESA prescription [14, 15]. Patients with a record of ESA prescription and ≥ 2 records of Hb < 10 or < 11 g/dL over 6 months were categorised as the ESA-treated cohort (referred to hereafter as Hb < 10 g/dL ESA-treated cohort and Hb < 11 g/dL ESA-treated cohort). ESAs were sub-categorised as short-acting (epoetin alfa/beta) or long-acting (darbepoetin alfa, methoxy polyethylene glycol-epoetin beta). Autoimmune diseases were defined by ICD-9-CM codes 696, 714, 720, 555, and 556 (see Online Resource 2 for further details). ICD-9-CM 250.0x (diabetes mellitus without mention of complication) and ATC A10 (drugs used in diabetes) codes were used as a proxy for diabetes.

Data analysis was primarily descriptive. Statistical significance was determined for binary variables using the Cochran–Armitage test, continuous variables using linear regression, and categorical variables using a Cochran–Mantel–Haenszel test. Analyses were reported by CKD stage as well as for the overall sample. All analyses were performed using STATA SE, version 12.0 (StataCorp LLC, College Station, TX, USA). End-stage renal disease (ESRD) risk assessment was determined using the Fine and Gray proportional sub-distribution hazards model, with death as a competing risk factor. Risk of death was determined using the Cox proportional hazards model. Covariates for each included: CKD stage, age, sex, cardiovascular disease, hypertension, diabetes mellitus, and autoimmune diseases.

## Results

### Disposition of patients

**Fig. 2:**
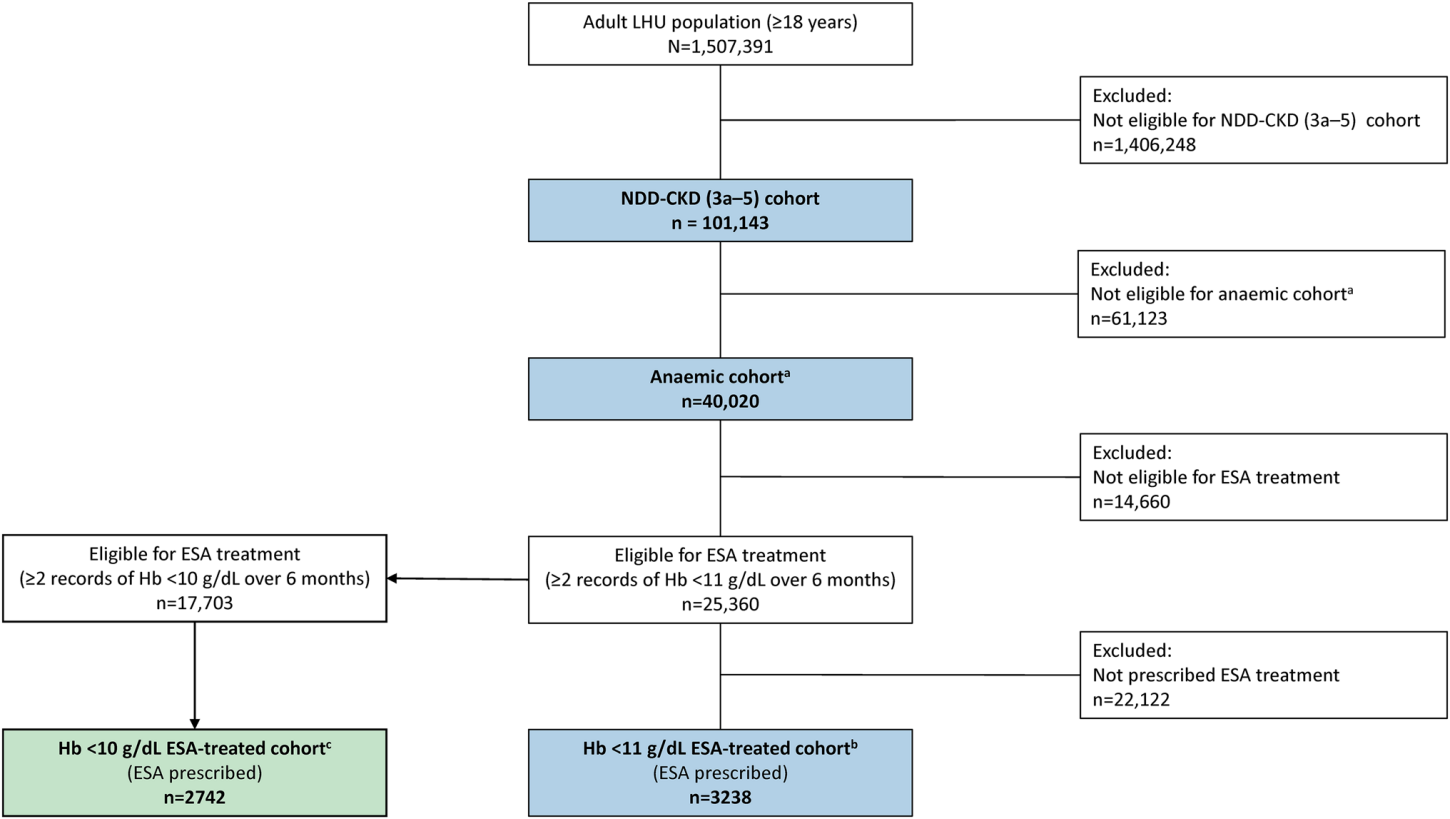
Patient flowchart. ^a^Anaemia of NDD-CKD (3a–5) cohort. ^b^Anaemia of NDD-CKD (3a–5) ESA-treated cohort (eligibility criterion for ESA treatment: ≥ 2 records of Hb < 11 g/dL over a 6-month period). ^c^Anaemia of NDD-CKD (3a–5) ESA-treated cohort (eligibility criterion for ESA treatment: ≥ 2 records of Hb < 10 g/dL over a 6-month period). *ESA* erythropoiesis-stimulating agent; *Hb* haemoglobin; *LHU* local health unit; *NDD-CKD* non-dialysis dependent chronic kidney disease

The five LHU databases included records for just over 1.5 million inhabitants, which corresponds to about 3% of the entire adult population of Italy [16]. A total of 101,143 individuals with NDD-CKD were included in the analysis, of whom 40,020 (39.6%) were anaemic (Fig. 2).

### Patient demographics and characteristics at index date

Over 70% of NDD-CKD patients had CKD stage 3a (Table 1). Mean age was 76.1 years and the majority of patients (> 90%) were aged ≥ 60 years. Approximately half of male patients were 60–79 years of age (50.7%), and approximately half of female patients were ≥ 80 years of age (50.2%) (Online Resource 4). No clear trends were observed for mean age across the CKD stages in the anaemic and ESA-treated cohorts (Online Resource 5 and 6). The proportion of males ranged between 39 and 48% across the CKD stages in the NDD-CKD cohort (Table 1). Of those patients with NDD-CKD, 43.3% of males and 37.0% of females were included in the anaemic cohort (Online Resource 4).

**Table 1:** Patient demographics for the NDD-CKD (3a–5) cohort at index date

	CKD stage				Overall	*p* value^d^
	3a	3b	4	5		
*N*	71,638	20,320	7189	1996	101,143	–
Age, mean (SD)	74.7 (12.4)	79.7 (11.2)	80.0 (12.1)	75.1 (14.5)	76.1 (12.4)	< 0.001
Age in years, *n* (%):						
18–39	948 (1.3)	139 (0.7)	68 (0.9)	40 (2.0)	1,195 (1.2)	< 0.001
40–59	6948 (9.7)	1036 (5.1)	439 (6.1)	245 (12.3)	8668 (8.6)	
60–79	35,632 (49.7)	7203 (35.4)	2284 (31.8)	788 (39.5)	45,907 (45.4)	
≥ 80	28,100 (39.2)	11,941 (58.8)	4397 (61.2)	920 (46.1)	45,358 (44.8)	
Male, *n* (%)	29,608 (41.3)	7939 (39.1)	2845 (39.6)	954 (47.8)	41,346 (40.9)	< 0.001
Included in anaemic cohort^a^, *n* (%)	23,626 (33.0)	10,181 (50.1)	4682 (65.1)	1531 (76.7)	40,020 (39.6)	–
Included in Hb < 11 g/dL ESA-treated cohort^b^, *n*	956 (1.3)	851 (4.2)	946 (13.2)	485 (24.3)	3238 (3.2)	–
Included in Hb < 10 g/dL ESA-treated cohort^c^, *n*	832 (1.2)	715 (3.5)	771 (10.7)	424 (21.2)	2742 (2.7)	–

Anaemia was defined as a Hb value below the cut-off specified by Kidney Disease Improving Global Outcomes (KDIGO) guidelines (< 13 g/dL in males and < 12 g/dL in females) [7]

*CKD* chronic kidney disease; *ESA* erythropoiesis-stimulating agent; *Hb* haemoglobin; *NDD-CKD* non-dialysis dependent chronic kidney disease; *SD* standard deviation

^a^Anaemia of NDD-CKD (3a–5) cohort

^b^Anaemia of NDD-CKD (3a–5) ESA-treated cohort (eligibility criterion for ESA treatment: ≥ 2 records of Hb < 11 g/dL over a 6-month period)

^c^Anaemia of NDD-CKD (3a–5) ESA-treated cohort (eligibility criterion for ESA treatment: ≥ 2 records of Hb < 10 g/dL over a 6-month period)

^d^Result of a statistical test to identify the existence of a trend in the ordered CKD stages

In general, rates of comorbidities tended to be slightly higher in the anaemic cohort than in the NDD-CKD cohort (Table 2 and Online Resource 7 and 8). Cardiovascular disease, hypertension, diabetes mellitus, autoimmune diseases, and myelodysplastic syndrome were detected in 23.5%, 89.9%, 32.4%, 2.1%, and 0.8% of patients with anaemia, respectively, without any apparent trends across the CKD stages (Table 2). The proportions of patients in the anaemic cohort who had received previous treatments for anaemia (ESAs, iron, blood transfusion) showed an upward trend with CKD progression (Table 2). Clinical characteristics of patients with anaemia of NDD-CKD stratified by sex can be found in Online Resource 9.

**Table 2:** Clinical characteristics of patients with anaemia of NDD-CKD (3a–5) at index date

	CKD stage				Overall	*p* value^a^
	3a	3b	4	5		
*N*	23,626	10,181	4682	1531	40,020	–
CV disease, *n* (%)	5321 (22.5)	2548 (25.0)	1216 (26.0)	328 (21.4)	9413 (23.5)	< 0.001
Hypertension, *n* (%)	20,827 (88.2)	9457 (92.9)	4322 (92.3)	1366 (89.2)	35,972 (89.9)	< 0.001
Diabetes mellitus, *n* (%)	7341 (31.1)	3451 (33.9)	1660 (35.5)	502 (32.8)	12,954 (32.4)	< 0.001
Autoimmune diseases, *n* (%)	513 (2.2)	211 (2.1)	97 (2.1)	30 (2.0)	851 (2.1)	0.886
Myelodysplastic syndrome, *n* (%)	196 (0.8)	84 (0.8)	49 (1.0)	9 (0.6)	338 (0.8)	0.416
Previous ESA treatment, *n* (%)	389 (1.6)	410 (4.0)	567 (12.1)	396 (25.9)	1762 (4.4)	< 0.001
Previous iron therapy (IV or oral), *n* (%)	211 (0.9)	175 (1.7)	200 (4.3)	136 (8.9)	722 (1.8)	< 0.001
Previous blood transfusion, *n* (%)	51 (0.2)	32 (0.3)	38 (0.8)	10 (0.7)	131 (0.3)	< 0.001

*CKD* chronic kidney disease; *CV* cardiovascular; *ESA* erythropoiesis-stimulating agent; *IV* intravenous

^a^Result of a statistical test to identify the existence of a trend in the ordered CKD stages

### Prevalence of NDD-CKD

In 2016, the standardised prevalence of NDD-CKD was 5.6% (Table 3). CKD stage 3a was the most common (4.2%), while the prevalence of stages 3b–5 ranged from 0.1–1.0%. Overall, there was a numerically higher proportion of female (6.3%) than male (4.7%) patients with NDD-CKD in 2016.

**Table 3:** Prevalence of NDD-CKD (3a–5) and anaemia of NDD-CKD (3a–5) in 2016

		CKD stage				Overall
		3a	3b	4	5	
Prevalence of NDD-CKD (3a–5) in the LHU patient population						
2016	Numerator (adult patients diagnosed)	62,683	15,221	4486	1235	83,625
2016	Denominator (adult LHU patient population)	1,507,391	1,507,391	1,507,391	1,507,391	1,507,391
** 2016**	**Prevalence**	**4.2%**	**1.0%**	**0.3%**	**0.1%**	**5.5%**
***2016***	***Standardised prevalence***^***a***^	***4.2%***	***1.0%***	***0.3%***	***0.1%***	***5.6%***
*2016*	*Standardised prevalence, male*	*3.5%*	*0.8%*	*0.3%*	*0.1%*	*4.7%*
*2016*	*Standardised prevalence, female*	*4.7%*	*1.2%*	*0.3%*	*0.1%*	*6.3%*
Prevalence of anaemia among patients with NDD-CKD (3a–5)						
2016	Numerator (patients diagnosed with anaemia)	17,681	6779	2826	973	28,259
2016	Denominator (NDD-CKD patients by CKD stage)	62,683	15,221	4486	1235	83,625
**2016**	**Prevalence**	**28.2%**	**44.5%**	**63.0%**	**78.8%**	**33.8%**
***2016***	***Standardised prevalence***^***a***^	***28.2%***	***44.6%***	***63.1%***	***78.9%***	***33.8%***
*2016*	*Standardised prevalence, male*	*31.2%*	*48.5%*	*66.7%*	*81.1%*	*37.0%*
*2016*	*Standardised prevalence, female*	*26.2%*	*42.2%*	*60.6%*	*76.5%*	*31.7%*

Bold italic text emphasizes the key data discussed in the body of the text rather than specific differences

*CKD* chronic kidney disease; *LHU* Local Health Unit; *NDD-CKD* non-dialysis dependent chronic kidney disease

^a^Prevalence at 31 December of each year was age- and sex-standardised using census data from 1 January of the following year

### Prevalence and incidence of anaemia of NDD-CKD

Standardised prevalence of anaemia among patients with NDD-CKD in 2016 was 33.8% (Table 3) and increased with CKD stage, rising from 28.2% among patients with stage 3a to 78.9% among those with stage 5. There was a numerically higher proportion of male (37.0%) than female (31.7%) patients with anaemia of NDD-CKD. Overall, the standardised incidence of anaemia of NDD-CKD was 12.4%, 12.3%, and 11.4% in 2014, 2015, and 2016, respectively, and increased with CKD stage within each year (Table 4). For all years, overall standardised incidence of anaemia of CKD was numerically higher for males than females.

**Table 4:** Annual incidence of anaemia among patients with NDD-CKD (3a–5) (2014–2016)

Year	Incidence of anaemia	CKD stage				Overall
		3a	3b	4	5	
2014	Numerator (patients newly diagnosed with anaemia)^a^	4174	2125	974	277	7550
2014	Denominator (NDD-CKD patients by CKD stage)	42,352	13,190	4472	1118	61,132
**2014**	**Incidence**	**9.9%**	**16.1%**	**21.8%**	**24.8%**	**12.4%**
***2014***	***Standardised incidence***^***b***^	***9.9%***	***16.1%***	***21.8%***	***24.8%***	***12.4%***
*2014*	*Standardised incidence, male*	*11.2%*	*17.9%*	*23.4%*	*23.6%*	*13.7%*
*2014*	*Standardised incidence, female*	*9.0%*	*15.1%*	*20.8%*	*25.9%*	*11.5%*
2015	Numerator (patients newly diagnosed with anaemia)^a^	5077	2078	807	248	8210
2015	Denominator (NDD-CKD patients by CKD stage)	50,131	12,339	3503	886	66,859
**2015**	**Incidence**	**10.1%**	**16.8%**	**23.0%**	**28.0%**	**12.3%**
***2015***	***Standardised incidence***^***b***^	***10.1%***	***16.9%***	***23.1%***	***28.0%***	***12.3%***
*2015*	*Standardised incidence, male*	*11.1%*	*18.3%*	*24.3%*	*26.6%*	*13.3%*
*2015*	*Standardised incidence, female*	*9.5%*	*16.0%*	*22.3%*	*29.6%*	*11.7%*
2016	Numerator (patients newly diagnosed with anaemia)^a^	4901	1823	702	225	7651
2016	Denominator (NDD-CKD patients by CKD stage)	52,945	10,788	2611	687	67,031
**2016**	**Incidence**	**9.3%**	**16.9%**	**26.9%**	**32.8%**	**11.4%**
***2016***	***Standardised incidence***^***b***^	***9.3%***	***16.9%***	***26.9%***	***32.8%***	***11.4%***
*2016*	*Standardised incidence, male*	10.3%	18.6%	29.6%	30.1%	12.5%
*2016*	*Standardised incidence, female*	8.6%	15.9%	25.1%	36.1%	10.7%
*p* value^c^		< 0.001	0.174	< 0.001	0.001	< 0.001

Bold italic text emphasizes the key data discussed in the body of the text rather than specific differences

*CKD* chronic kidney disease; *NDD-CKD* non-dialysis dependent chronic kidney disease

^a^Incident cases were defined as newly diagnosed NDD-CKD during the year of interest

^b^Annual incidence was age- and sex-standardised using census data from 1 January of the following year

^c^*p* value refers to the comparison of standardised incidence value among different years

### Eligibility for ESAs and ESA treatment

Using the Hb < 11 g/dL criterion to determine ESA eligibility, 25,360 patients were considered eligible for ESA treatment, of whom 3238 were prescribed ESAs. Using the Hb < 10 g/dL criterion, 17,703 patients were considered eligible for ESA treatment, and 2742 were prescribed ESAs (Fig. 2). Over the whole study period (2014–2016), the overall proportion of patients with anaemia of NDD-CKD who were eligible for ESA treatment increased with CKD stage, regardless of the eligibility criterion used, or sex (Table 5; Online Resource 10). The proportion of ESA-eligible patients was 63.4% using the Hb < 11 g/dL threshold (57.6% for male and 68.0% for female patients), and 44.2% using the Hb < 10 g/dL threshold (40.7% for male and 47.1% for female patients). There was an upward trend across the CKD stages for the proportion of eligible patients who were treated with ESAs. The proportion of eligible patients treated with ESAs was 12.8% when the Hb threshold was < 11 g/dL, ranging from 6.7% for patients in stage 3a to 39.8% for those in stage 5. Using the stricter eligibility criterion of Hb < 10 g/dL, a slightly higher proportion of eligible patients were treated with ESAs overall (15.5%) and for each CKD stage.

**Table 5:** Proportion of patients with anaemia of NDD-CKD (3a–5) eligible for ESA treatment, and treated with ESAs (overall study period, 2014–2016)

	CKD stage				Overall	*p* value^a^
	3a	3b	4	5		
Number of patients with anaemia of NDD-CKD (3a–5)	23,626	10,181	4682	1531	40,020	–
Eligibility criterion ≥ 2 records of Hb < 11 g/dL over a 6-month period						
Eligible for ESA, *n* (%)	14,203 (60.1)	6592 (64.7)	3347 (71.5)	1218 (79.6)	25,360 (63.4)	< 0.001
Eligible patients treated with ESAs, *n*/*N* (%)	956/14,203 (6.7)	851/6592 (12.9)	946/3347 (28.3)	485/1218 (39.8)	3238/25,360 (12.8)	< 0.001
Eligibility criterion ≥ 2 records of Hb < 10 g/dL over a 6-month period						
Eligible for ESA, *n* (%)	9675 (41.0)	4612 (45.3)	2442 (52.2)	974 (63.6)	17,703 (44.2)	< 0.001
Eligible patients treated with ESAs, *n*/*N* (%)	832/9675 (8.6)	715/4612 (15.5)	771/2442 (31.6)	424/974 (43.5)	2742/17,703 (15.5)	< 0.001

*CKD* chronic kidney disease; *ESA* erythropoiesis-stimulating agent; *Hb* haemoglobin; *NDD-CKD* non-dialysis dependent chronic kidney disease

^a^Result of statistical test to identify the existence of a trend in the ordered CKD stages

Overall, 59.5% (1927/3238) of patients in the Hb < 11 g/dL ESA-treated cohort received short-acting ESAs and 49.3% (1597/3238) received long-acting ESAs. The corresponding proportions for the Hb < 10 g/dL ESA-treated cohort were 62.0% (1699/2742) and 47.4% (1300/2742), respectively. Some patients switched between the two ESA types, receiving both long- and short-acting ESAs during the study period. In the Hb < 11 g/dL ESA-treated cohort, the proportion of patients receiving short-acting ESAs decreased with CKD stage from 69.6% to 54.0%, in favour of long-acting ESAs (the proportion of patients increased from 36.1% for stage 3a to 63.7% for stage 5). A similar pattern was observed for the Hb < 10 g/dL ESA-treated cohort.

### Iron supplementation and blood transfusions (patients with at least 2 years of follow up)

Intravenous (IV) iron infusions were received by 9.0% of patients in the anaemic cohort, and 18.4% and 19.3% of patients in the Hb < 11 g/dL ESA-treated and Hb < 10 g/dL ESA-treated cohorts, respectively (Table 6). Data for patients who received oral iron during the follow-up period were not consistently available in the study databases, and thus were not analysed here. Blood transfusions were received by 11.3%, 16.5%, and 19.4% of patients in the anaemic cohort, Hb < 11 g/dL ESA-treated cohort, and Hb < 10 g/dL ESA-treated cohort, respectively.

**Table 6:** Proportions of patients in the anaemia of NDD-CKD (3a–5) and ESA-treated cohorts with at least 2 years of follow up who received intravenous iron supplementation and blood transfusions

	CKD stage				Overall	*p* value^e^
	3a	3b	4	5		
Intravenous iron^a^, *n*/*N* (%)						
Anaemic cohort^b^	1564/16,509 (9.5)	472/6336 (7.4)	196/2528 (7.8)	108/658 (16.4)	2340/26,031 (9.0)	< 0.001
Hb < 11 g/dL ESA-treated cohort^c^	127/590 (21.5)	67/547 (12.2)	82/515 (15.9)	64/192 (33.3)	340/1844 (18.4)	< 0.001
Hb < 10 g/dL ESA-treated cohort^d^	113/495 (22.8)	56/439 (12.8)	68/392 (17.3)	49/155 (31.6)	286/1481 (19.3)	< 0.001
Blood transfusion, *n*/*N* (%)						
Anaemic cohort^b^	1803/16,509 (10.9)	728/6336 (11.5)	334/2528 (13.2)	81/658 (12.3)	2946/26,031 (11.3)	0.026
Hb < 11 g/dL ESA-treated cohort^c^	123/590 (20.8)	83/547 (15.2)	79/515 (15.3)	19/192 (9.9)	304/1844 (16.5)	0.014
Hb < 10 g/dL ESA-treated cohort^d^	118/495 (23.8)	79/439 (18.0)	72/392 (18.4)	19/155 (12.3)	288/1481 (19.4)	0.054

Percentages calculated using the proportion of patients with at least 2 years of follow up in each cohort

Anaemia was defined as a Hb value below the cut-off specified by Kidney Disease Improving Global Outcomes (KDIGO) guidelines (< 13 g/dL in males and < 12 g/dL in females) [7]

CKD, chronic kidney disease; ESA, erythropoiesis-stimulating agent; Hb, haemoglobin; NDD-CKD, non-dialysis dependent chronic kidney disease

^a^No data were available for oral iron therapy

^b^Anaemia of NDD-CKD (3a–5) cohort

^c^Anaemia of NDD-CKD (3a–5) ESA-treated cohort (eligibility criterion for ESA treatment: ≥ 2 records of Hb < 11 g/dL over a 6-month period)

^d^Anaemia of NDD-CKD (3a–5) ESA-treated cohort (eligibility criterion for ESA treatment: ≥ 2 records of Hb < 10 g/dL over a 6-month period)

^e^ Result of statistical test to identify the existence of a trend in the ordered CKD stages

### Risk of end stage renal disease and death

The sub-distribution model showed that CKD stage, age, sex, hypertension, diabetes mellitus, and anaemia had a significant effect on the development of ESRD in patients with CKD (*p < *0.001 for all; Online Resource 11). In particular, anaemia was associated with a fourfold higher risk of ESRD (sub-distribution hazard ratio 4.00, confidence interval [CI]: 2.75–5.81; *p < *0.001). Similarly, we found an increased risk of death in CKD patients with anaemia (hazard ratio 2.24, CI: 2.17–2.31; *p < *0.001) compared to patients without anaemia.

## Discussion

To date, this is the largest real-world study on anaemia of NDD-CKD in Italy. Starting with records for approximately 1.5 million inhabitants from a pool of five areas geographically distributed across Italy, 101,143 patients with NDD-CKD were identified. Anaemia of NDD-CKD was characterised in terms of epidemiology, demographics, clinical characteristics, and management.

The overall standardised prevalence of NDD-CKD in 2016 was 5.6%. Previous Italian studies describing prevalence rates for CKD have not reported data on anaemia, or focused on NDD-CKD, and used different patient inclusion criteria; therefore, those findings are not directly comparable with the results from this research [2, 17]. In this study, the standardised prevalence rate in 2016 for anaemia among patients with NDD-CKD was 33.8%, which differs from rates (41–62%) reported by other Italian studies enrolling patients regularly followed in renal clinics [11, 12]. Different prevalence rates reported by studies are expected given the variations in study populations and methodology. Patients with NDD-CKD were predominantly female in the present study. This is consistent with previous studies, which report that the prevalence of CKD was higher in female patients, despite increased severity and risk of progression of CKD in male patients [18]. In the present study, the prevalence of anaemia among patients with NDD-CKD was numerically higher in males than females, which conflicts with findings from previous studies that females were more likely to develop anaemia of CKD than males [3, 19]. This may be a consequence of the higher Hb cut-off value in male patients compared to females (< 13 g/dL versus < 12 g/dL) for the definition of anaemia in the present study. As a result, the Hb cut-off value might be attained at an earlier stage of disease in male than female patients [20]. Furthermore, comorbidities with the potential to induce anaemia (e.g., CV disease and diabetes) were reported in higher proportions of male patients than female patients in this study. Standardised prevalence and incidence of anaemia among patients with NDD-CKD increased through CKD stages, suggesting a greater need for anaemia management in patients with more advanced CKD. Trends in the standardized incidence of anaemia among patients with NDD-CKD from 2014 to 2016, overall and by CKD stage, were variable; particularly for stage 3a where anaemia was less frequent.

Anaemia is known to become more common as CKD progresses, and may be associated with increased risks of cardiovascular disease, hospitalisation, cognitive impairment, and death [6]. In the current study, hypertension was the most frequent comorbidity, with rates of 83.8% to 96.4% across the three cohorts, and without a clear relationship with CKD stage. These observations are in line with other studies in patients with NDD-CKD, in which about 80–87% of patients had hypertension [21, 22]. Similarly, no trend across CKD stages was observed for the prevalence of diabetes mellitus, a clinical condition more frequently associated with new-onset anaemia [23]. The present study also showed that a diagnosis of anaemia in patients with CKD was associated with a higher risk of ESRD, and of death, independently of risk factors related to adverse outcomes in this patient population [11, 22, 24, 25].

Among anaemic patients, we applied two different cut-offs of Hb concentration (≥ 2 records of either Hb < 10 or < 11 g/dL over 6 months) in estimating ESA eligibility to reflect international guidelines and clinical practice in Italy, respectively. This ensured that the data would be relevant worldwide and not just in Italy. Requiring ≥ 2 records over 6 months rather than a single record reduced the chance that a reduction in Hb may have had a temporary cause such as inflammation, infection, bleeding, or surgery [26]. Based on the two eligibility criteria, 44.2% (Hb < 10 g/dL) and 63.4% (Hb < 11 g/dL) of anaemic patients were eligible for ESA treatment. Regardless of the Hb cut-off, the proportion of eligible patients actually treated with ESAs was low (≤ 15.5%), although the proportion treated increased across the CKD stages (from < 10% in stage 3a to approximately 40% in stage 5). These results suggest that patients who required treatment for anaemia were inadequately treated when ESAs could have been prescribed. Therapeutic inertia has been reported previously in the context of nephrology care, with 34% of NDD-CKD patients not receiving ESAs despite having anaemia over a 6-month observation period [12]. This phenomenon has been confirmed recently by the CKDopps analysis, which showed that a large proportion of patients with anaemia of NDD-CKD did not receive anaemia medication within 1 year [10]. However, it would be an oversimplification to conclude from the present study that anaemia was undertreated. Indeed, international guidelines recommend that the decision to treat with ESAs is not based on Hb level alone, but should also consider symptoms related to anaemia, prior cardiovascular history (e.g., stroke), active or past history of malignancy, rate of decrease of Hb concentration, prior response to iron therapy, the risk for transfusion, and risks related to ESA therapy [7, 26]. Therefore, the large proportion of untreated patients may reflect the need for healthcare professionals to understand the underlying causes of anaemia before prescribing ESAs, while also reducing the burden of cost of inappropriate prescriptions on the National Health Service. In addition, this large untreated patient population may rely on a sub-optimal healthcare service, which may not refer all patients to the appropriate specialist, such as a nephrologist, to receive treatment. Of note, the present analysis was based on ESAs prescribed to patients in the outpatient setting, and ESA use during hospitalisation was not captured. However, ESAs prescribed during hospitalisation continue to be given after discharge, and therefore the absence of hospital records of ESA use is unlikely to have affected the study findings.

The proportion of anaemic patients who received IV iron infusions in the present study (9.0%) was generally consistent with data reported in other studies. A study using data from the CKDopps found that IV iron was received by 12%, 9%, 33%, and 8% of patients with anaemia of CKD stages 3a–5 in Brazil, France, Germany, and the USA, respectively [9]. Another study conducted in Italy found that IV iron was prescribed to approximately 3% of NDD-CKD patients receiving iron supplements [12]. A challenge remains in the estimation of the proportion of patients receiving oral iron therapy. Oral iron supplements are likely to be the predominant form of iron therapy in patients with NDD-CKD, but their over-the-counter availability makes their use more difficult to track. As oral iron data were not readily available in the databases used for the current study, these data were not analysed for the follow-up period.

An original finding of this study was the rate of blood transfusions in the NDD-CKD population. Studies from the USA have reported a rising prevalence of blood transfusions in the non-dialysis population, which has been linked with a concomitant and marked reduction in the use of ESAs [27]. This greater use of blood transfusions than ESAs or IV iron is believed to have been triggered by policy changes, including drug reimbursement, and additional FDA ‘black box’ warnings related to the potential adverse effects of ESAs [27]. Indeed, in one US study, the proportion of NDD-CKD patients aged 66–85 years with anaemia who received blood transfusions (22.2%) was found to be almost double the proportion observed in anaemic patients (11.3%) in the present study [27]. This finding could be explained by differences between the US and Italian healthcare systems. Of note, in this study the use of blood transfusions decreased with advancing CKD stage in parallel with increasing ESA prescriptions. This trend was independent of the severity of anaemia (either Hb < 10 g/dL or < 11 g/dL).

The ‘real-world’ setting and large sample size are major strengths of this study. Using population-based data, a ‘snapshot’ has been provided of the management of anaemia in more than 100,000 patients with NDD-CKD in Italy, a population for whom these epidemiologic data were lacking. This study, however, has limitations. We cannot exclude that other haemoglobinopathies (in particular thalassaemia) have been classified as renal anaemia in our study, although the advanced age of our study population strongly limits the potential impact of thalassaemia on our estimates. Also, the inclusion of patients with anaemia from non-CKD-related causes in this study may have resulted in a slight overestimation of the number of patients eligible for ESAs, and the occurrence of clinical inertia. Frequently, the use of large administrative databases to investigate a specific clinical question can be associated with limited suitable data, thus reducing cohort size [28]. The databases captured only direct medical healthcare resource use reimbursed by the Italian National Health Service. In patients without ICD-9-CM codes for CKD, only one creatinine value was used to define the presence of CKD, whereas international guidelines require at least two pathological creatinine values or other markers of kidney damage more than 3 months apart [29]. This discrepancy may have resulted in a partial overestimation of CKD cases. A proxy (e.g., specific drugs, hospitalisations) was used for comorbidities, such as diabetes, which may have introduced error in estimating their prevalence.

The length of look-back period can also impact estimates of prevalence and incidence: a shorter look-back period can potentially lead to the overestimation of an incidence rate due to the misclassification of prevalent and recurrent cases as incident cases [30]. The minimum look-back period for inclusion in this study was 1 year but could have been up to 8 years depending on the individual patient’s inclusion date; however, the length of look-back period was not analysed for this study. Other limitations include the evaluation of drug treatments based on prescriptions only, and therefore ESA prescriptions could have been related to conditions other than anaemia of CKD. Patients with a cancer diagnosis were excluded from the study in order to reduce this potential bias, as ESAs could be prescribed to these patients and therefore could have influenced the results for CKD and anaemia. Eligibility for ESA treatment was based on Hb levels alone. As other decision criteria for ESA treatment recommended by the guidelines, such as symptoms of anaemia and prior cardiovascular history, were not recorded in the LHU databases, it is not possible to comment on the extent to which patients with anaemia may have been undertreated [7, 26]. The iron supplementation data were limited to IV iron. Lastly, it is unknown to what extent the data for Italy from the current study may be generalised to other countries given the differences in healthcare systems.

In conclusion, this study demonstrated that anaemia is a significant issue in patients with NDD-CKD, and the incidence increases with CKD stage. The proportion of patients eligible for ESA treatment who actually received ESAs was relatively low, indicating a potential treatment gap, and suggesting that anaemia may not be adequately controlled. There appears to be an unmet need to remedy the apparent clinical inertia and improve the diagnosis and treatment of anaemia of NDD-CKD in Italy. However, this study also highlights the careful choices made by healthcare professionals when prescribing ESAs, accounting for both the underlying conditions of the patients, and the cost of inappropriate ESA prescription to the Italian National Health Service.

## Supplementary Information

Below is the link to the electronic supplementary material.

Supplementary file1 (DOCX 87 kb)

## Data Availability

Researchers may request access to anonymised participant level data, trial level data, and protocols from Astellas-sponsored clinical trials at www.clinicalstudydatarequest.com. For the Astellas criteria on data sharing see: https://clinicalstudydatarequest.com/Study-Sponsors/Study-Sponsors-Astellas.aspx.

## Declarations

### Previous presentation

These data were presented in part as a mini-oral presentation at the 58th European Renal Association – European Dialysis and Transplant Association (ERA-EDTA) Annual Congress, 5–8 June 2021, Berlin, Germany.
