# Supplementary material for: Prevalence, incidence, and treatment of anaemia in patients with non-dialysis-dependent chronic kidney disease: findings from a retrospective real-world study in Italy
Source: J Nephrol. 2022 Nov 12;36(2):347–57. doi: 10.1007/s40620-022-01475-x (PMC9998309; doi:10.1007/s40620-022-01475-x)
Supplement: Supplementary file 1 — Supplementary file1 (DOCX 87 kb) [file 40620_2022_1475_MOESM1_ESM.docx]

**SUPPLEMENTARY INFORMATION**

**Title**: Prevalence, incidence, and treatment of anaemia in patients with non-dialysis dependent chronic kidney disease: findings from a retrospective real-world study in Italy

**Authors**: Roberto Minutolo^1^, Giuseppe Grandaliano^2,3^, Paolo Di Rienzo^4^, Robert Snijder^5^, Luca Degli Esposti^6^, Valentina Perrone^6^, Lora Todorova^7^

**Target journal**: *The Journal of Nephrology*

**Corresponding author**:

Name: Roberto Minutolo

Address: Department of Advanced Medical and Surgical Sciences, University of Campania Luigi Vanvitelli, Naples, Italy

Email: roberto.minutolo@unicampania.it

## Online Resource 1 Details of ethics committees

| **Local Health Unit** | **Ethics Committee** | **Reference number** | **Approval date** |
| --- | --- | --- | --- |
| Vercelli | COMITATO ETICO INTERAZIENDALE | Prot. N AslVC.Farm.19.02 | 19/12/2019 |
| Barletta Andria Trani | COMITATO ETICO INTERPROVINCIALE AREA 1 | Prot. 96/SegCE/2019 | 03/12/2019 |
| Roma 6 | COMITATO ETICO LAZIO 2 | Prot. N. 0219118 | 11/12/2019 |
| Teramo | COMITATO ETICO PER LE PROVINCE DI L’AQUILA E TERAMO | Prot. N. 03 | 30/01/2020 |
| Trieste | COMITATO ETICO UNICO REGIONALE DEL FRIULI VENEZIA GIULIA | Prot. CEUR-2020-Os-029 | 28/01/2020 |

**Online Resource 2** Supplementary Methods

### Data sources

Data were extracted from administrative (demographic, hospitalisation, pharmaceutical, and outpatient specialist services) and laboratory databases of five Local Health Units (LHUs) across Italy (Barletta Andria Trani, Roma 6, Vercelli, Teramo, and Trieste). These databases are used by the Italian National Health System for the reimbursement of healthcare services. To guarantee patients’ privacy, an anonymous univocal numeric code was assigned to each patient included in the study, in full compliance with the European General Data Protection Regulation (GDPR) (2016/679) [1]. This code allowed the electronic linkage of all the databases. No identifiers related to patients were provided to the authors. All the results of the analyses were produced as aggregated summaries, which were not possible to assign, either directly or indirectly, to individual patients. Informed consent was not required, in accordance with authorisation no. 9/2014 of the Italian Data Protection Authority [2], as collection of consent was impossible for organisational reasons. According to Italian law regarding the conduct of observational studies, the Ethics Committee of each participating LHU was notified of this study and provided approval (**Online Resource 1**).

Data extracted from the databases and used in this analysis included chronic kidney disease (CKD) stage, laboratory tests (haemoglobin, creatinine), drug prescriptions, blood transfusions, hospitalisation (with associated diagnosis-related group), and demographic and clinical characteristics.

### Definitions of non-dialysis dependent (NDD)-CKD and CKD stage

NDD-CKD (3a–5) was defined as either ≥1 hospitalisation record with a diagnosis of CKD (ICD-9-CM 585.x, where x = 3, 4, or 5) or ≥1 record of estimated glomerular filtration rate (eGFR) <60 mL/min/1.73 m^2^. Classification of CKD stage (3a–5) based on eGFR was done according to Kidney Disease Improving Global Outcomes (KDIGO) guidelines [3]. eGFR was derived from creatinine laboratory test results using the Modification of Diet in Renal Disease (MDRD) method.

*Description of ICD-9 codes*

ICD-9-CM codes used in this study to define autoimmune diseases were: 696 (psoriasis and similar disorders), 714 (rheumatoid arthritis and other inflammatory polyarthropathies), 720 (ankylosing spondylitis and other inflammatory spondylopathies), 555 (regional enteritis), and 556 (idiopathic proctocolitis).

### Comorbidities

Comorbidities were identified either by hospitalisation discharge ICD-9-CM codes or by the presence of prescription for specific treatments identified from pharmaceutical databases through Anatomical Therapeutic Chemical (ATC) codes and considered as a proxy for diagnosis.

## References

1. Official Journal of the European Union (2016) Regulation (EU) 2016/679 of the European Parliament and of the Council of 27 April 2016 on the protection of natural persons with regard to the processing of personal data and on the free movement of such data, and repealing Directive 95/46/EC. http://data.europa.eu/eli/reg/2016/679/oj. Accessed 2 July 2021

2. Garante per la Protezione dei Dati Personali (2014) Authorisation no. 9/2014 - general authorisation to process personal data for scientific research purposes [3786078]. https://www.garanteprivacy.it/home/docweb/-/docweb-display/docweb/3786078. Accessed 2 July 2021

3. Kidney Disease: Improving Global Outcomes (KDIGO) (2013) KDIGO 2012 clinical practice guideline for the evaluation and management of chronic kidney disease. Kidney Int Suppl 3:4477–4783. doi:10.1038/ki.2013.243

## Online Resource 3 Incident and prevalent patient cohorts

| **Patient cohort** | **Definition** |
| --- | --- |
| Incident NDD-CKD | Patients whose CKD started upon their inclusion in the study: no record of CKD during the period preceding their inclusion in the study |
| Prevalent NDD-CKD | Patients who already had CKD before their inclusion in the study or were incident: at least one record of CKD during the period preceding their inclusion in the study or during the inclusion period |
| Incident anaemia of NDD-CKD | NDD-CKD patients whose anaemia started upon their inclusion in the study: no record of anaemia during the period preceding their inclusion in the study |
| Prevalent anaemia of NDD-CKD | NDD-CKD patients who were already anaemic before their inclusion in the study or were incident: at least one record of anaemia during the period preceding their inclusion in the study or during the inclusion period |
| Incident ESA-treated | Patients with anaemia of NDD-CKD whose earliest ESA prescription was during the study inclusion period: no record of ESA treatment during the period preceding their inclusion in the study |
| Prevalent ESA-treated | Patients with anaemia of NDD-CKD whose earliest ESA prescription was prior to their inclusion in the study or were incident: at least one record of ESA treatment during the year preceding their inclusion in the study or during the inclusion period |
| CKD, chronic kidney disease; ESA, erythropoiesis-stimulating agent; NDD-CKD, non-dialysis dependent chronic kidney disease. | |

**Online Resource 4** Patient demographics for the NDD-CKD (3a–5) cohort at index date, stratified by gender

|  | **CKD stage** | | | | **Overall** | **p-value^d^** |
| --- | --- | --- | --- | --- | --- | --- |
|  | **3a** | **3b** | **4** | **5** |  |  |
| **Male patients in the NDD-CKD (3a–5) cohort** | | | | | | |
| N | 29,608 | 7939 | 2845 | 954 | 41,346 | – |
| Age, mean (SD) | 72.9 (12.4) | 77.1 (11.5) | 76.8 (12.7) | 72.2 (14.1) | 74.0 (12.4) | <0.001 |
| Age in years, n (%): |  |  |  |  |  |  |
| 18–39 | 449 (1.5) | 63 (0.8) | 35 (1.2) | 21 (2.2) | 568 (1.4) | <0.001 |
| 40–59 | 3446 (11.6) | 611 (7.7) | 264 (9.3) | 148 (15.5) | 4469 (10.8) |  |
| 60–79 | 16,007 (54.1) | 3378 (42.5) | 1138 (40.0) | 459 (48.1) | 20,982 (50.7) |  |
| ≥80 | 9701 (32.8) | 3887 (49.0) | 1408 (49.5) | 326 (34.2) | 15,322 (37.1) |  |
| Included in anaemic cohort^a^, n (%) | 10,844 (36.6) | 4335 (54.6) | 1957 (68.8) | 767 (80.4) | 17,903 (43.3) | <0.001 |
| Included in Hb <11 g/dL ESA-treated cohort^b^, n (%) | 526 (1.8) | 428 (5.4) | 446 (15.7) | 253 (26.5) | 1653 (4.0) | <0.001 |
| Included in Hb <10 g/dL ESA-treated cohort^c^, n (%) | 469 (1.6) | 351 (4.4) | 364 (12.8) | 215 (22.5) | 1399 (3.4) | <0.001 |
| **Female patients in the NDD-CKD (3a–5) cohort** | | | | | |  |
| N | 42,030 | 12,381 | 4344 | 1042 | 59,797 | – |
| Age, mean (SD) | 75.9 (12.3) | 81.4 (10.6) | 82.0 (11.3) | 77.7 (14.5) | 77.5 (12.2) | <0.001 |
| Age in years, n (%): |  |  |  |  |  |  |
| 18–39 | 499 (1.2) | 76 (0.6) | 33 (0.8) | 19 (1.8) | 627 (1.0) | <0.001 |
| 40–59 | 3502 (8.3) | 425 (3.4) | 175 (4.0) | 97 (9.3) | 4199 (7.0) |  |
| 60–79 | 19,625 (46.7) | 3825 (30.9) | 1146 (26.4) | 329 (31.6) | 24,925 (41.7) |  |
| ≥80 | 18,399 (43.8) | 8054 (65.1) | 2989 (68.8) | 594 (57.0) | 30,036 (50.2) |  |
| Included in anaemic cohort^a^, n (%) | 12,782 (30.4) | 5846 (47.2) | 2725 (62.7) | 764 (73.3) | 22,117 (37.0) | <0.001 |
| Included in Hb <11 g/dL ESA-treated cohort^b^, n (%) | 430 (1.0) | 423 (3.4) | 500 (11.5) | 232 (22.3) | 1585 (2.7) | <0.001 |
| Included in Hb <10 g/dL ESA-treated cohort^c^, n (%) | 363 (0.9) | 364 (2.9) | 407 (9.4) | 209 (20.1) | 1343 (2.2) | <0.001 |

Anaemia was defined as a Hb value below the cut-off specified by Kidney Disease Improving Global Outcomes (KDIGO) guidelines (<13 g/dL in males and <12 g/dL in females) [7].

^a^Anaemia of NDD-CKD (3a–5) cohort.
^b^Anaemia of NDD-CKD (3a–5) ESA-treated cohort (eligibility criterion for ESA treatment: ≥2 records of Hb <11 g/dL over a 6-month period).
^c^Anaemia of NDD-CKD (3a–5) ESA-treated cohort (eligibility criterion for ESA treatment: ≥2 records of Hb <10 g/dL over a 6-month period).

^d^Result of statistical test to identify the existence of a trend in the ordered CKD stages.

CKD, chronic kidney disease; ESA, erythropoiesis-stimulating agent; Hb, haemoglobin; NDD-CKD, non-dialysis dependent chronic kidney disease; SD, standard deviation.

## Online Resource 5 Patient demographics in the anaemia of NDD-CKD (3a–5) cohort at index date

|  | **CKD stage** | | | | **Overall** | **p-value**^a^ |
| --- | --- | --- | --- | --- | --- | --- |
|  | **3a** | **3b** | **4** | **5** |  |  |
| N | 23,626 | 10,181 | 4682 | 1531 | 40,020 | – |
| Age, mean (SD) | 78.5 (11.1) | 81.0 (10.5) | 80.1 (11.9) | 74.8 (14.1) | 79.2 (11.2) | <0.001 |
| Age in years, n (%): |  |  |  |  |  |  |
| 18–39 | 182 (0.8) | 55 (0.6) | 41 (0.9) | 34 (2.2) | 312 (0.8) | <0.001 |
| 40–59 | 1210 (5.2) | 383 (3.8) | 255 (5.4) | 179 (11.8) | 2027 (5.1) |  |
| 60–79 | 9730 (42.6) | 3227 (32.7) | 1521 (32.9) | 633 (41.4) | 15,111 (38.9) |  |
| ≥80 | 12,501 (51.4) | 6516 (62.9) | 2865 (60.7) | 685 (44.5) | 22,567 (55.2) |  |
| Male, n (%) | 10,844 (45.9) | 4335 (42.6) | 1957 (41.8) | 767 (50.1) | 17,903 (44.7) | <0.001 |
| ^a^Result of statistical test to identify the existence of a trend in the ordered CKD stages.  CKD, chronic kidney disease; SD, standard deviation. | | | | | |  |

## Online Resource 6 Patient demographics in the anaemia of NDD-CKD (3a–5) ESA-treated cohort at index date

|  | **CKD stage** | | | | **Overall** | **p-value**^c^ |
| --- | --- | --- | --- | --- | --- | --- |
|  | **3a** | **3b** | **4** | **5** |  |  |
| **Hb <11 g/dL ESA-treated^a^** | | | | | |  |
| N | 956 | 851 | 946 | 485 | 3,238 | – |
| Age, mean (SD) | 76.6 (10.9) | 79.7 (10.6) | 77.4 (12.5) | 71.6 (14.3) | 76.9 (12.1) | <0.001 |
| Age in years, n (%): |  |  |  |  |  |  |
| 18–39 | 9 (0.9) | 8 (0.9) | 14 (1.8) | 11 (2.5) | 42 (1.4) | <0.001 |
| 40–59 | 55 (5.9) | 34 (4.5) | 65 (7.2) | 84 (17.5) | 238 (7.6) |  |
| 60–79 | 461 (50.5) | 303 (38.5) | 374 (41.8) | 230 (47.4) | 1368 (44.3) |  |
| ≥80 | 430 (42.6) | 506 (56.1) | 493 (49.3) | 160 (32.6) | 1589 (46.6) |  |
| Male, n (%) | 526 (55.0) | 428 (50.3) | 446 (47.1) | 253 (52.2) | 1653 (51.1) |  |
| **Hb <10 g/dL ESA-treated^b^** | | | | | |  |
| N | 832 | 715 | 771 | 424 | 2,742 | – |
| Age, mean (SD) | 76.8 (10.6) | 79.9 (10.7) | 77.2 (12.6) | 72.0 (14.1) | 77.0 (12.0) | <0.001 |
| Age in years, n (%): |  |  |  |  |  |  |
| 18–39 | 5 (0.6) | 7 (1.0) | 12 (1.9) | 10 (2.6) | 34 (1.4) | <0.001 |
| 40–59 | 47 (5.8) | 30 (4.5) | 54 (7.4) | 67 (15.8) | 198 (7.4) |  |
| 60–79 | 403 (50.6) | 246 (38.0) | 304 (41.5) | 204 (48.3) | 1157 (44.4) |  |
| ≥80 | 376 (42.9) | 432 (56.5) | 401 (49.2) | 143 (33.3) | 1352 (46.7) |  |
| Male, n (%) | 469 (56.4) | 351 (49.1) | 364 (47.2) | 215 (50.7) | 1399 (51.0) | <0.001 |
| ^a^Anaemia of NDD-CKD (3a–5) ESA-treated cohort (eligibility criterion for ESA treatment: ≥2 records of Hb <11 g/dL over a 6-month period). ^b^Anaemia of NDD-CKD (3a–5) ESA-treated cohort (eligibility criterion for ESA treatment: ≥2 records of Hb <10 g/dL over a 6-month period).  ^C^Result of statistical test to identify the existence of a trend in the ordered CKD stages.  CKD, chronic kidney disease; ESA, erythropoiesis-stimulating agent; SD, standard deviation. | | | | | |  |

**Online Resource 7** Clinical characteristics of patients in the NDD-CKD (3a–5) cohort at index date

|  | **CKD stage** | | | | **Overall** | **p-value**^a^ |
| --- | --- | --- | --- | --- | --- | --- |
|  | **3a** | **3b** | **4** | **5** |  |  |
| N | 71,638 | 20,320 | 7189 | 1996 | 101,143 |  |
| CV disease, n (%) | 11,239 (15.7) | 4296 (21.1) | 1751 (24.4) | 410 (20.5) | 17,696 (17.5) | <0.001 |
| Hypertension, n (%) | 60,022 (83.8) | 18,640 (91.7) | 6594 (91.7) | 1750 (87.7) | 87,006 (86.0) | <0.001 |
| Diabetes, n (%) | 17,983 (25.1) | 6188 (30.5) | 2429 (33.8) | 635 (31.8) | 27,235 (26.9) | <0.001 |
| Autoimmune diseases, n (%) | 1153 (1.6) | 329 (1.6) | 125 (1.7) | 35 (1.8) | 1642 (1.6) | 0.825 |
| Myelodysplastic syndrome, n (%) | 263 (0.4) | 101 (0.5) | 59 (0.8) | 9 (0.5) | 432 (0.4) | <0.001 |
| ^a^Result of statistical test to identify the existence of a trend in the ordered CKD stages.  CKD, chronic kidney disease; CV, cardiovascular; NDD-CKD, non-dialysis dependent chronic kidney disease. | | | | | |  |

## Online Resource 8 Clinical characteristics of patients in the anaemia of NDD-CKD (3a–5) ESA-treated cohort at index date

|  | **CKD stage** | | | | **Overall** | **p-value**^c^ |
| --- | --- | --- | --- | --- | --- | --- |
|  | **3a** | **3b** | **4** | **5** |  |  |
| **Hb <11 g/dL ESA-treated^a^** | | | | | |  |
| N | 956 | 851 | 946 | 485 | 3238 |  |
| CV disease, n (%) | 196 (20.5) | 246 (28.9) | 269 (28.4) | 118 (24.3) | 829 (25.6) | <0.001 |
| Hypertension, n (%) | 868 (90.8) | 811 (95.3) | 912 (96.4) | 454 (93.6) | 3045 (94.0) | <0.001 |
| Diabetes, n (%) | 361 (37.8) | 346 (40.7) | 409 (43.2) | 188 (38.8) | 1304 (40.3) | <0.001 |
| Autoimmune diseases, n (%) | 28 (2.9) | 20 (2.4) | 17 (1.8) | 8 (1.6) | 73 (2.3) | 0.363 |
| **Hb <10 g/dL ESA-treated^b^** | | | | | |  |
| N | 832 | 715 | 771 | 424 | 2742 |  |
| CV disease, n (%) | 165 (19.8) | 216 (30.2) | 225 (29.2) | 109 (25.7) | 715 (26.1) | <0.001 |
| Hypertension, n (%) | 752 (90.4) | 682 (95.4) | 741 (96.1) | 396 (93.4) | 2571 (93.8) | <0.001 |
| Diabetes, n (%) | 308 (37.0) | 296 (41.4) | 332 (43.1) | 166 (39.2) | 1102 (40.2) | <0.001 |
| Autoimmune diseases, n (%) | 22 (2.6) | 16 (2.2) | 16 (2.1) | 8 (1.9) | 62 (2.3) | 0.858 |
| ^a^Anaemia of NDD-CKD (3a–5) ESA-treated cohort (eligibility criterion for ESA treatment: ≥2 records of Hb <11 g/dL over a 6-month period). ^b^Anaemia of NDD-CKD (3a–5) ESA-treated cohort (eligibility criterion for ESA treatment: ≥2 records of Hb <10 g/dL over a 6-month period).  ^C^Result of statistical test to identify the existence of a trend in the ordered CKD stages.  CKD, chronic kidney disease; CV, cardiovascular; ESA, erythropoiesis-stimulating agent; NR, not reported (in accordance with ‘Opinion 05/2014 on Anonymisation Techniques’ drafted by the ‘European Commission Article 29 Working Party’, analyses involving ≤3 patients were not reported). | | | | | |  |

## Online Resource 9 Clinical characteristics of patients with anaemia of NDD-CKD (3a–5) at index date, stratified by gender

|  | **CKD stage** | | | | **Overall** |
| --- | --- | --- | --- | --- | --- |
|  | **3a** | **3b** | **4** | **5** |  |
| **Male patients with anaemia of NDD-CKD (3a–5)** | | | | | |
| N | 10,844 | 4335 | 1957 | 767 | 17,903 |
| CV disease, n (%) | 2928 (27.0) | 1286 (29.7) | 566 (28.9) | 175 (22.8) | 4955 (27.7) |
| Hypertension, n (%) | 9477 (87.4) | 3959 (91.3) | 1770 (90.4) | 666 (86.8) | 15,872 (88.7) |
| Diabetes mellitus, n (%) | 3629 (33.5) | 1532 (35.3) | 710 (36.3) | 257 (33.5) | 6128 (34.2) |
| Autoimmune diseases, n (%) | 189 (1.7) | 87 (2.0) | 36 (1.8) | 12 (1.6) | 324 (1.8) |
| Previous ESA treatment, n (%) | 208 (1.9) | 206 (4.8) | 249 (12.7) | 190 (24.8) | 853 (4.8) |
| Previous iron therapy (IV or oral), n (%) | 97 (0.9) | 87 (2.0) | 87 (4.4) | 66 (8.6) | 337 (1.9) |
| Previous blood transfusion, n (%) | 31 (0.3) | 15 (0.3) | 15 (0.8) | 4 (0.5) | 65 (0.4) |
| **Female patients with anaemia of NDD-CKD (3a–5)** | | | | | |
| N | 12,782 | 5846 | 2725 | 764 | 22,117 |
| CV disease, n (%) | 2393 (18.7) | 1262 (21.6) | 650 (23.9) | 153 (20.0) | 4458 (20.2) |
| Hypertension, n (%) | 11,350 (88.8) | 5498 (94.0) | 2552 (93.7) | 700 (91.6) | 20,100 (90.9) |
| Diabetes mellitus, n (%) | 3712 (29.0) | 1919 (32.8) | 950 (34.9) | 245 (32.1) | 6826 (30.9) |
| Autoimmune diseases, n (%) | 324 (2.5) | 124 (2.1) | 61 (2.2) | 18 (2.4) | 527 (2.4) |
| Previous ESA treatment, n (%) | 181 (1.4) | 204 (3.5) | 318 (11.7) | 206 (27.0) | 909 (4.1) |
| Previous iron therapy (IV or oral), n (%) | 114 (0.9) | 88 (1.5) | 113 (4.1) | 70 (9.2) | 385 (1.7) |
| Previous blood transfusion, n (%) | 20 (0.2) | 17 (0.3) | 23 (0.8) | 6 (0.8) | 66 (0.3) |
| CKD, chronic kidney disease; CV, cardiovascular; ESA, erythropoiesis-stimulating agent; IV, intravenous. | | | | | |

## Online Resource 10 Proportion of male and female patients with anaemia of NDD-CKD (3a–5) eligible for ESA treatment, and treated with ESAs (overall study period, 2014–2016)

|  | **CKD stage** | | | | **Overall** | **p-value^a^** |
| --- | --- | --- | --- | --- | --- | --- |
|  | **3a** | **3b** | **4** | **5** |  |  |
| **Male patients with anaemia of NDD-CKD (3a–5)** | | | | |  |  |
| N | 10,844 | 4335 | 1957 | 767 | 17,903 |  |
| Hb <11 g/dL ESA-treated^b^ | | | | | |  |
| Eligible for ESA, n (%) | 5846 (53.9) | 2547 (58.8) | 1308 (66.8) | 612 (79.8) | 10313 (57.6) | <0.001 |
| Eligible patients treated with ESAs, n/N (%) | 526/5846 (9.0) | 428/2547 (16.8) | 446/1308 (34.1) | 253/612 (41.3) | 1653/10,313 (16.0) | <0.001 |
| Hb <10 g/dL ESA-treated^c^ | | | | | |  |
| Eligible for ESA, n (%) | 4049 (37.3) | 1808 (41.7) | 939 (48.0) | 482 (62.8) | 7278 (40.7) | <0.001 |
| Eligible patients treated with ESAs, n/N (%) | 469/4049 (11.6) | 351/1808 (19.4) | 364/939 (38.8) | 215/482 (44.6) | 1399/7278 (19.2) | <0.001 |
| **Female patients with anaemia of NDD-CKD (3a–5)** | | | | | | |
| N | 12,782 | 5846 | 2725 | 764 | 22,117 |  |
| Hb <11 g/dL ESA-treated^b^ | | | | | | |
| Eligible for ESA, n (%) | 8357 (65.4) | 4045 (69.2) | 2039 (74.8) | 606 (79.3) | 15047 (68.0) | <0.001 |
| Eligible patients treated with ESAs, n/N (%) | 430/8357 (5.1) | 423/4045 (10.5) | 500/2039 (24.5) | 232/606 (38.3) | 1585/15,047 (10.5) | <0.001 |
| Hb <10 g/dL ESA-treated^c^ | | | | | | |
| Eligible for ESA, n (%) | 5626 (44.0) | 2804 (48.0) | 1503 (55.2) | 492 (64.4) | 10,425 (47.1) | <0.001 |
| Eligible patients treated with ESAs, n/N (%) | 363/5626 (6.5) | 364/2804 (13.0) | 407/1503 (27.1) | 209/492 (42.5) | 1343/10,425 (12.9) | <0.001 |
| ^a^Result of statistical test to identify the existence of a trend in the ordered CKD stages.  ^b^Anaemia of NDD-CKD (3a–5) ESA-treated cohort (eligibility criterion for ESA treatment: ≥2 records of Hb <11 g/dL over a 6-month period). ^c^Anaemia of NDD-CKD (3a–5) ESA-treated cohort (eligibility criterion for ESA treatment: ≥2 records of Hb <10 g/dL over a 6-month period).  CKD, chronic kidney disease; ESA, erythropoiesis-stimulating agent; Hb, haemoglobin; NDD-CKD, non-dialysis dependent chronic kidney disease. | | | | | |  |

## Online Resource 11 Multivariable models; risk of ESRD and death in patients with CKD.

|  | **ESRD (competing risk: death)** | | | | **Death** | | | |
| --- | --- | --- | --- | --- | --- | --- | --- | --- |
|  |  |  |  |  |  |  |  |  |
| ESRD | SHR | 95% CI | | p-value | HR | 95% CI | | p-value |
| CKD stage |  |  |  |  |  |  |  |  |
| 3a | REF. |  |  |  | REF. |  |  |  |
| 3b | 2.913 | 1.909 | 4.446 | <0.001 | 1.373 | 1.327 | 1.421 | <0.001 |
| 4 | 9.067 | 6.098 | 13.481 | <0.001 | 1.930 | 1.846 | 2.017 | <0.001 |
| 5 | 54.921 | 37.566 | 80.294 | <0.001 | 2.507 | 2.327 | 2.701 | <0.001 |
| Age | 0.946 | 0.938 | 0.953 | <0.001 | 1.072 | 1.070 | 1.074 | <0.001 |
| Male | 1.869 | 1.436 | 2.432 | <0.001 | 1.288 | 1.250 | 1.327 | <0.001 |
| CV disease | 1.075 | 0.796 | 1.452 | 0.637 | 1.267 | 1.224 | 1.310 | <0.001 |
| Hypertension | 2.279 | 1.413 | 3.675 | 0.001 | 0.774 | 0.737 | 0.814 | <0.001 |
| Diabetes mellitus | 1.432 | 1.103 | 1.857 | 0.007 | 1.012 | 0.979 | 1.045 | 0.483 |
| Autoimmune diseases | 0.815 | 0.425 | 1.564 | 0.539 | 1.123 | 1.003 | 1.259 | 0.045 |
| Anaemia | 4.001 | 2.754 | 5.812 | <0.001 | 2.236 | 2.166 | 2.308 | <0.001 |

ESRD risk assessment was determined using the Fine and Gray proportional sub-distribution hazards model, with death as a competing risk factor. Risk of death was determined using the Cox proportional hazards model.
ESRD, end stage renal disease; CKD, chronic kidney disease; CV, cardiovascular; HR, hazard ratio; SHR, sub-distribution hazard ratio.
